# Weight Gain and Hair Loss during Anti-TNF Therapy

**DOI:** 10.1155/2012/593039

**Published:** 2012-08-05

**Authors:** Abdo Lutf, Mohammed Hammoudeh

**Affiliations:** Rheumatology Division, Department of Medicine, Hamad General Hospital, P.O. Box 3050, Doha, Qatar

## Abstract

*Objectives*. To investigate the incidence of weight gain and hair loss as adverse effects of anti-TNF therapy in rheumatic diseases. *Methods*. Patients using anti-TNF therapy, who are followed in rheumatology clinic, were interviewed using a questionnaire to investigate the side effects of anti-TNF therapy. Patients who complained of hair loss and weight gain were asked additional questions concerning the relationship of these adverse effects to anti-TNF use, whether therapy was stopped because of these adverse effects and if the adverse effects reversed after stopping therapy. The files were reviewed to follow the weight change before, during, and after discontinuation of anti-TNF. *Results*. One hundred fifty consecutive patients (82 RA, 34 ankylosing spondylitis, 32 psoriatic arthritis, and 4 for other indications) were interviewed .Weight gain was observed in 20 patients (13.3%) with average gain of 5.5 Kg. Anti-TNF was stopped in five patients because of this adverse effect. Hair loss during anti-TNf therapy was reported in five females (3.3%) and anti-TNF therapy was stopped in all of them. *Conclusion*. Weight gain and hair loss appear to be associated with anti-TNF therapy and may be one reason for discontinuing the therapy.

## 1. Introduction

Decrease in lean mass can occur in patients with chronic advanced diseases and has been described in chronic inflammatory diseases such as Rheumatoid Arthritis (RA), where it may result in profound weight loss [1]. These changes are linked to the effects of cytokines such as TNF-*α* and interleukins and also the level of disease activity.

 In recent years, a growing number of biological agents have been introduced for the treatment of various diseases one of them is anti-TNF therapy which is approved for the treatment of many patients with inflammatory arthritis who fail to gain satisfactory disease control with conventional disease-modifying antirheumatic drugs (DMARDs). In randomized clinical trials (RCTs) all of these agents have been shown to be effective in reducing clinical signs of inflammation in RA, spondyloarthropathy.

 Adverse effects of anti-TNF therapy include activation of infections and reactivation of latent TB. Less critical adverse effects are weight gain which is rarely reported in small observational studies in RA and spondyloarthropathy [2–5] and hair loss (alopecia areata or generalized alopecia) which has been reported as case report mainly in psoriatic patients on anti-TNF therapy [6, 7]. These effects may be of great concern to some patients, especially females, and despite the benefits of these therapies, the adverse effects may lead to discontinuation of anti-TNF therapy.

 We conducted this retrospective study in Hamad Medical Corporation to find out the incidence of these adverse effects in our patients on anti-TNF, and how often a decision is taken to discontinue treatment because of these adverse effects.

## 2. Methods

All patients on anti-TNF treatment for rheumatic diseases that are being followed in our rheumatology clinic in Hamad Medical Corporation were interviewed by the authors. Data collected included age, gender, name of the present, and previous anti-TNFs; patients were asked whether they had gained weight ordeveloped hair loss while on anti-TNF and if a decision had been made to discontinue the anti-TNF therapy because of these adverse effects.

Hair loss as a side effect of biologics was considered if the patient developed it after starting the anti-TNF, the dose of methotrexate had not been increased during that period and there was no other reason to explain it. The patient's file was reviewed to verify the documentation of the hair loss and if discontinuation of the anti-TNF was due to hair loss in the opinion of the physician.

Weight gain was considered as an adverse effect if it was reported by the patient and it was noticed by the patient after starting anti-TNF therapy and there was no other obvious reason for it. The files of patients with weight gain were reviewed to determine the timing and the degree of weight gain after starting anti-TNF therapy and if the weight gain had resulted in discontinuation of the anti-TNF.

## 3. Results

Of 150 of patients on anti-TNF (82 RA, 34 ankylosing spondylitis, 32 psoriatic arthritis, and 4 for other indications), 20 (13.3%) cases were reported weight gain in first year while on anti-TNF (18 females and two males). The range of weight gain was from 2.2 kg to 12 kg with an average weight gain of 5.5 kg. The pretreatment average of BMI was 31.2 and after treatment was 35.8. Anti-TNF was discontinued in 5/20 (25%) due to this adverse effects. Ten patients were on etanrecept, eight on adalimumab, and four on infliximab. Two of the twenty patients developed weight gain on two consecutive anti-TNF infliximab and adalimumab. Glucocorticoids were used in 6/20 of our patients in dose (5–10 mg of prednisolone) and was started before anti-TNF.

Hair loss after using anti-TNF was reported in five female (4 SPA, 1 RA) patients (3.3%); anti-TNF was stopped in all of them. Two were on etanrecept and two on adalimumab one on infliximab.

 Hair loss improved after discontinuation of anti-TNF in all patients.

## 4. Discussion

A significant number of our patients on anti-TNF (13.3%) developed weight gain with an average of 5.5 kg (average BMI changes 4.7) and it led to discontinuation of anti-TNf in 25% of them, representing 3.3% of all the patients on anti-TNF therapy. Though the incidence of hair loss was less than weight gain (3.3%) all of the affected patients were females and the anti-TNF was discontinued in them due to this adverse effect.

These drugs have been shown to be effective in reducing clinical signs of inflammation in RA, SPA, and effective in reducing radiographic progression [8–10] and seem to be safe and well-tolerated drugs but with their increasing use and longer follow-up periods of treatment, a spectrum of adverse events including increased risk of infections, TB reactivation, and new immune-mediated diseases have been observed such as lupus-like syndrome, systemic lupus erythematosus (SLE), and interstitial lung disease [11].

Weight gain and hair loss have been reported as side effects of anti-TNF in several publications. Alcorn et al. investigated body composition in 20 patients with RA after 12 weeks of treatment with anti-TNF therapy but did not observe any change in weight or fat-free mass [3]. However after 12 months it was shown that of 53 RA patients receiving anti-TNF therapy, 73% gained weight during the first 12 months of treatment (mean, 4.17; range, 0.5–13 kg) [3]. A very recent study investigated the effects of infliximab on body composition in a randomized study with 25 patients [4], an increase in fat mass was observed in those patients randomized to infliximab after 21 months of treatment but not in those patients randomized to DMARDs alone. Weight gain has also been observed in a number of chronic inflammatory conditions other than RA, which have been treated with anti-TNF therapy. In a study of 106 patients with ankylosing spondylitis (AS), a significant increase in weight was observed after 12 months of treatment with anti-TNF therapy (2.2 ± 3.9 kg), which remained elevated at 24 months [5].

Hair loss (Alopecia areata) has been reported in many cases [6, 7]. Ferran et al. from Spain reported five cases of alopecia areata (AA) and reviewing the literature they found 11 cases of AA induced by anti-TNF-*α* therapies [6].

This data showed that weight gain and hair loss associated with anti-TNF therapy are not rare side effects and may result in discontinuation of anti-TNF. Further prospective studies are needed.
